# Limited SUMOylation inhibitor administration enhances eradication of Burkitt’s lymphoma with CD19 CAR-T therapy

**DOI:** 10.1038/s41392-025-02422-5

**Published:** 2025-10-03

**Authors:** Hiroshi Kotani, Shigeki Sato, Seiji Yano, Marco L. Davila, Hiroaki Taniguchi

**Affiliations:** 1 Kanazawa University, Kanazawa, Japan; 2 Roswell Park Comprehensive Cancer Center, Buffalo, NY USA

**Keywords:** Drug development, Haematological cancer

**Dear Editor**,

Burkitt’s lymphoma (BL) is a highly aggressive B-cell lymphoma and one of the fastest-growing tumors, driven by hallmark *MYC* oncogene translocation. While BL is often curable with high-dose chemotherapy, relapsed/refractory cases have a poor prognosis, even with CD19-targeted chimeric antigen receptor T-cell (CAR-T) therapy^1^ or immune checkpoint inhibitors. Therefore, BL is considered a cold tumor that may be triggered by MYC dysregulation.

Inspired by our recent studies that a SUMOylation inhibitor TAK-981 downregulated MYC protein expression via proteasomal degradation and also modulated antitumor immune responses,^2,3^ we examined the efficacy of TAK-981 in human BL cell lines and *Eμ-Myc* mouse-derived cell line. Cell viability assay revealed high sensitivity to TAK-981 (IC_50_ < 1 μM) in all cell lines (Fig. 1a). Moreover, we observed that downregulation of MYC and apoptosis represented by upregulation of cleaved-PARP were induced in cells treated with 100 nM TAK-981 for 48 h (Fig. 1a). Therefore, we manifested that TAK-981 shut down the critical pathway in BL cells.

**Fig. 1:**
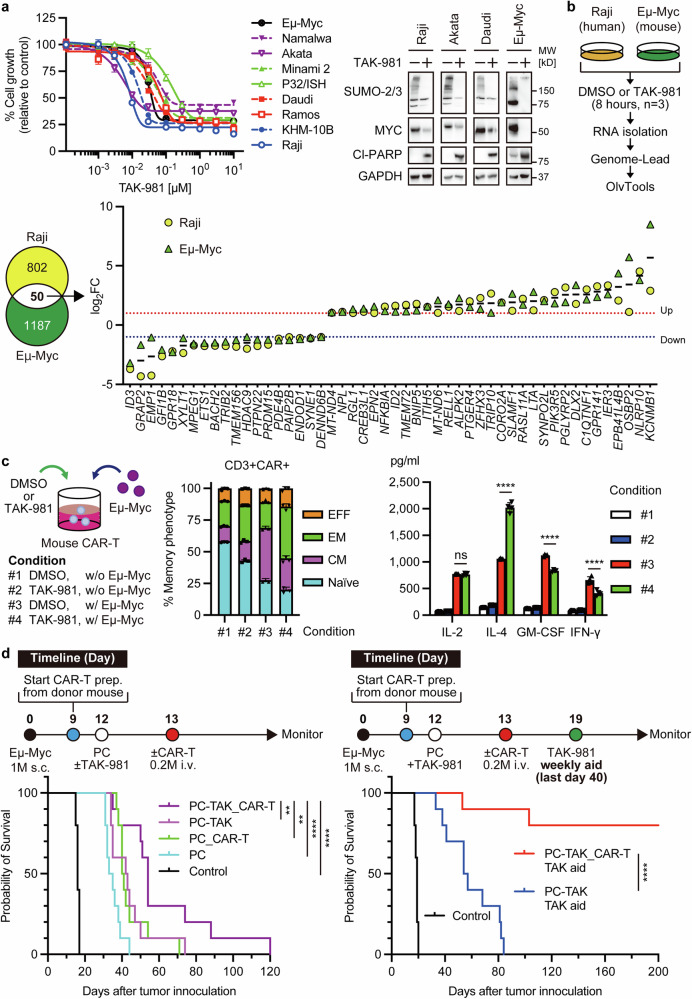
Enhancing the curability of Burkitt’s lymphoma with CD19 CAR-T therapy via limited SUMOylation inhibitor administration. **a** *Left*: Sensitivity of human and mouse cell lines to TAK-981. Cells were treated with nine concentrations of TAK-981 (threefold serial dilutions from 10 µM to 1.5 nM) or 0.1% DMSO for 72 h (*n* = 6 per dose). *Right*: Immunoblotting of cells treated with 100 nM TAK-981 for 48 h. **b** *Up*: Identification of TAK-981-modulated genes in Raji and Eμ-Myc cells. Cells were treated with 0.1% DMSO or 100 nM TAK-981 for 8 h before RNA isolation. Triplicate RNA-seq samples were processed by Genome-Lead and analyzed using OlvTools. *Down-Left*: Venn diagram depicting differentially expressed genes (DEGs) in Raji, Eμ-Myc. *Down-Right*: Visualization of common DEGs. **c** *Left*: In vitro culture conditions for CAR-T experiments. 2.5 × 10^5^ CAR-T cells were cultured for 24 h under four conditions: #1 0.1% DMSO, #2 100 nM TAK-981, #3 2.5 × 10^5^ Eμ-Myc cells, #4 2.5 × 10^5^ Eμ-Myc cells + 100 nM TAK-981. Biological quadruplicates were analyzed. *Center*: Flow cytometry immunophenotyping of mouse T cells. *Right*: Representative Bio-Plex Pro Mouse Cytokine 23-Plex Assay data. **d** *Left*: Efficacy of TAK-981 combined with conventional preconditioning chemotherapy (PC) in a syngeneic immunocompetent high-tumor-burden Eμ-Myc Burkitt’s lymphoma model. Tumors reached around 400 mm^3^ on day 12. Treatment groups; Control: No treatment (*n* = 5), PC: Cyclophosphamide (200 mg/kg) + fludarabine (20 mg/kg) i.p. (day 12) (*n* = 10), PC-TAK: PC + TAK (25 mg/kg) i.p. (on day 12) (n = 10), PC_CAR-T: PC (day 12) + 2 × 10^5^ CAR-T i.v. (day 13) (*n* = 10), PC-TAK_CAR-T: PC + TAK-981 (day 12) + 2 × 10^5^ CAR-T (day 13) (*n* = 10). Mice were monitored until tumor growth/recurrence ( > 1000 mm^3^) or health deterioration. **p < 0.01, ****p < 0.0001 (Log-rank test). *Right*: Efficacy of limited TAK-981 aid post-CAR-T therapy. Treatment groups; Control: No treatment (*n* = 5), PC-TAK + TAK-aid: PC + TAK-981 (day 12) + TAK-981 on days 19, 26, 33, 40 (*n* = 10), PC-TAK_CAR-T TAK aid: PC + TAK-981 (day 12) + 2 × 10^5^ CAR-T (day 13) + TAK-981 on days 19, 26, 33, 40 (*n* = 10). ****p < 0.0001 (Log-rank test)

To investigate how SUMOylation inhibition early affects global gene transcription, we performed gene expression profiling to examine transcriptional changes after TAK-981 treatment. Human-derived Raji and mouse-derived Eμ-Myc cells were treated with DMSO or 100 nM TAK-981 for 8 h and then analyzed by RNA-seq (Fig. 1b). Although numerous transcriptional changes ( > 2-fold) were observed in volcano plots (data not shown), the common differentially expressed genes were 50 genes. Interestingly, downregulation of *ID3* was identified, which may explain escape signals from the inhibition of critical pathway-associated MYC because *ID3* is also one of the key regulators of cell proliferation in BL cells (Fig. 1b). On the other hand, upregulation of *NLRP10* was identified, which suggested the stimulation of innate immunity. It can endorse our recent study.^3^ Given the efficacy of TAK-981 in vitro, we performed an in vivo experiment using an Eμ-Myc subcutaneous syngeneic immunocompetent mouse model. However, TAK-981 monotherapy twice a week exhibited modest efficacy in the model, starting with an average tumor volume of 140 mm^3^ (data not shown).

Next, we sought to determine whether TAK-981 enables the enhancement of CAR-T efficacy because CD19 CAR-T therapy does not satisfy treatment in the clinical study.^1^ To perform CD19 CAR-T related experiments, we used GFP-tagged second-generation mouse CAR harboring mouse CD19-targeted single chain variable fragment paired to mouse CD8α hinge and transmembrane domain, mouse CD28 co-stimulatory domain, and CD3ζ domain.^4^ We checked the influences of CAR-positive and negative mouse T cells in vitro in the following conditions: (#1) CAR-T cells in DMSO control, (#2) CAR-T cells in 100 nM TAK-981, (#3) CAR-T cells co-cultured with Eμ-Myc cells at an E:T ratio of 1:1 in DMSO control, or (#4) CAR-T cells co-cultured with Eμ-Myc cells at an E:T ratio of 1:1 in 100 nM TAK-981 (Fig. 1c). First, we compared immunophenotypes of the cells after 24 h exposure of them. While the proportion of effector phenotype of CAR negative T cells modestly increased by Eμ-Myc exposure (conditions #3 and #4), the memory phenotypes of CAR-T cells were largely affected by TAK-981 or/and Eμ-Myc exposure (Fig. 1c). TAK-981 (#2 and #4) promoted effector-like phenotypes, while Eμ-Myc exposure (#3 and #4) shifted CAR-T cells toward naïve-like to central memory phenotypes. Effector-like T cells have limited self-renewal capacity compared to naïve-like or central memory phenotypes, suggesting prolonged TAK-981 treatment could reduce CAR-T persistence in vivo. Second, we demonstrated a multiplex suspension array to compare cytokine secretion by CAR-T cells using the supernatants above. CAR-T cells secreted multiple cytokines in co-culture with Eμ-Myc cells (#3 and #4) (Fig. 1c). Interestingly, higher IL-4 and lower GM-CSF & IFN-γ levels were detected in #4 compared to #3, although both IL-2 levels were similar. These results suggested that TAK-981 polarized CAR-T cells to the Th2 subtype when CAR-T cells encountered the antigen and may enhance cytotoxicity.^5^ Third, we assessed the cytotoxicity of mouse CAR-T cells using 3T3-mCD19-MYC cells (artificial antigen-presenting cells overexpressing MYC) in the presence or absence of TAK-981 via real-time cell analysis (RTCA). Because the RTCA instrument primarily measures adherent cells, we generated MYC-overexpressing 3T3-mCD19 cells. As anticipated based on our previous findings,^2^ 3T3-mCD19-MYC cells showed enhanced sensitivity to TAK-981 compared to parental 3T3-mCD19 cells (data not shown). In addition, enhanced cytotoxicity was observed treated with mouse CAR-T cells and TAK-981 (data not shown).

Then, we performed an in vivo study using the Eμ-Myc mouse model. To start with, we conducted the CAR-T treatment following preconditioning chemotherapy (PC) of cyclophosphamide and fludarabine or PC adding on TAK-981 only once (PC-TAK) in a low tumor burden model. Interestingly, all mice survived for a long period in both ways (data not shown). Next, we sought to determine whether the high-tumor-burden model mimicking a clinical situation exhibited the same results. Although PC-TAK prolonged the survival of mice compared to conventional PC, all mice were deceased for long-term follow-up (Fig. 1d). To address treatment failure, we modified the regimen by adding four weekly doses of TAK-981 (TAK aid). Surprisingly, 80% of mice treated with PC-TAK, CAR-T, and TAK aid survived for the long term (Fig. 1d). These results strongly suggested that limited addition of SUMOylation inhibition to CAR-T boosted curable treatment in BL. In addition, we also performed for human CAR-T cells using 3T3-hCD19-MYC as above and confirmed the concordance between mouse and human CAR-T cells’ cytotoxicity (data not shown). While the cytotoxicity under E:T ratio 1:1 condition regardless of treatment with/without TAK-981 was equivalent, the cytotoxicity under E:T ratio 1:5 condition was stronger in the presence of TAK-981 treatment than that without TAK-981 treatment. Collectively with the results of different tumor burden mouse models, TAK-981 may play important roles to eradicate BL. However, since we have not evaluated human CAR-T in vivo models, further investigations are warranted.

In summary, we discovered that limited administration of SUMOylation inhibitor could eradicate BL treated with CD19 CAR-T. Direct tumor growth suppression and Th2-polarized CAR-T via SUMOylation inhibition may shine a light on the BL treatment strategy in the future.

## Supplementary information

Supplementary Information

## Data Availability

RNA-seq data before and after TAK-981 treatment of Raji and Eμ-Myc have been submitted to the BIG Sub with the BioProject ID PRJCA045094.
