# Supplementary material for: Limited SUMOylation inhibitor administration enhances eradication of Burkitt’s lymphoma with CD19 CAR-T therapy
Source: Signal Transduct Target Ther. 2025 Oct 3;10:325. doi: 10.1038/s41392-025-02422-5 (PMC12491439; doi:10.1038/s41392-025-02422-5)
Supplement: Supplementary file 1 — Supplementary Information [file 41392_2025_2422_MOESM1_ESM.docx]

Supplementary Information for

Limited SUMOylation inhibitor administration enhances eradication of Burkitt’s lymphoma with CD19 CAR-T therapy

Hiroshi Kotani, Shigeki Sato, Seiji Yano, Marco L Davila, and Hiroaki Taniguchi

Correspondence to: h.kotani@staff.kanazawa-u.ac.jp

**This PDF file includes:**

Materials and Methods

Materials and Methods

**Reagents**

TAK-981 was purchased from Selleck Chemicals. RPMI-1640, IMDM, DMEM, fetal bovine serum (FBS), penicillin/streptomycin, and L-glutamine were obtained from Gibco. Sodium pyruvate and MEM non-essential amino acids (NEAA) were purchased from Wako. HEPES and β-mercaptoethanol were acquired from Sigma-Aldrich.

**Cells**

Akata, Daudi, KHM-10B, Minami 2, Namalwa, P32/ISH, Raji, and Ramos cell lines were obtained from JCRB and cultured in RPMI-1640 supplemented with 10% heat-inactivated FBS, 100 U/mL penicillin, and 100 µg/mL streptomycin. Eμ-Myc, H29, Phoenix E, RD114, 3T3-mCD19 and 3T3-hCD19 cell lines were provided by the Davila lab (Roswell Park Comprehensive Cancer Center). Eμ-Myc cells were cultured with irradiated (30 Gy) NIH/3T3 fibroblasts (ATCC) as feeders. The Eμ-Myc culture medium consisted of equal volumes of: (1) IMDM supplemented with 10% heat-inactivated FBS, 2 mM L-glutamine, 55 µM β-mercaptoethanol, 100 U/mL penicillin, and 100 µg/mL streptomycin, and (2) DMEM supplemented with 10% heat-inactivated FBS, 2 mM L-glutamine, 100 U/mL penicillin, and 100 µg/mL streptomycin. H29, Phoenix E, RD114, 3T3-mCD19 and 3T3-hCD19 cells were cultured in DMEM supplemented with 10% heat-inactivated FBS, 100 U/mL penicillin, and 100 µg/mL streptomycin. 3T3-mCD19 and 3T3-hCD19 cells were NIH/3T3 cells retrovirally transduced with mouse or human CD19 as previously reported.^1^ For 3T3-mCD19-MYC and 3T3-hCD19-MYC cells, MYC expression plasmid DNA was transfected into 3T3-mCD19 and 3T3-hCD19 cells using Lipofectamine 3000 Transfection Reagent (Thermo Fisher Scientific) as previously reported.^2^ Mouse T cell complete medium consisted of RPMI-1640 medium, 10% heat-inactivated FBS, 1 mM sodium pyruvate, 1× MEM NEAA, 10 mM HEPES, 55 μM β-mercaptoethanol, 2 mM L-glutamine, 100 U/mL penicillin, and 100 μg/mL streptomycin. Human peripheral blood mononuclear cells (PBMCs) from a healthy donor were purchased form Precision for Medicine. Human T cell complete medium consisted of RPMI-1640 medium, 10% heat-inactivated FBS, 2 mM L-glutamine, 100 U/mL penicillin, and 100 μg/mL streptomycin. All cell lines were regularly screened for mycoplasma contamination using the MycoAlert Mycoplasma Detection Kit (Lonza). Cell number and viability were determined using a LUNA-FX7 (Logos Biosystems).

**Cell Viability Assay**

Cells were seeded in 96-well plates and treated with 0.1% DMSO or nine different concentrations of TAK-981 (3-fold serial dilution from 10 µM, n=6). After 72 hours, Cell Counting Kit-8 (Dojindo) was added, and cell viability was determined by measuring absorbance. Data were visualized using GraphPad Prism 9.

**Immunoblot Analysis**

Cell lysates were collected using CelLytic M (Sigma-Aldrich) supplemented with 1% phosphatase inhibitor cocktail 3 (Sigma-Aldrich) and 10 µM phenylmethanesulfonyl fluoride (Sigma-Aldrich). Immunodetection was performed using standard protocols, and signals were detected using a Chemiluminescence Imaging System (M&S Instruments Inc.). The following antibodies were used: anti-SUMO-2/3 (#4971), anti-MYC (#13987), anti-Cleaved-PARP (#5625), anti-GAPDH (#5174), and HRP-linked anti-rabbit IgG (#7074) (all from Cell Signaling Technology).

**Gene Expression Analysis**

RNA was extracted using the RNeasy Plus Kit (QIAGEN) according to the manufacturer’s instructions and stored at -80ºC. Samples were shipped to Genome-Lead (Japan), enriched by PolyA, and sequenced using DNBSEQ (MGI Tech). FASTQ data were analyzed and visualized using OlvTools.

**Genetic Constructs and CAR-T Cell Production**

The SFG retroviral construct for m1928z CAR tagged with GFP has been described previously.^1^ The SFG retroviral construct for h1928z CAR tagged with mCherry was generated by VectorBuilder. The SFG constructs were transfected into H29 cells using calcium phosphate. Retroviral supernatants from transfected H29 cells were harvested and used to transduce Phoenix E cells for mouse T cell transduction or RD114 cells for human T cell transduction. Retroviral supernatant from Phoenix E or RD114 producer cells was harvested, filtered (0.45-μm), and used to transduce mouse or human T cells. To produce mouse CAR-T cells, T cells were isolated from the spleens of C57BL/6 (B6) mice (Charles River Laboratories Japan) using the EasySep Mouse T cell Isolation Kit (STEMCELL Technologies). To produce human CAR-T cells, T cells were isolated from a healthy donor’s PBMCs using the EasySep Human T cell Isolation Kit (STEMCELL Technologies). T cells were activated with Dynabeads Mouse or Human T-Activator CD3/CD28 (Gibco) on day 1, according to the manufacturers’ instruction. On days 2 and 3, T cells were retrovirally transduced with CAR via spinoculation on RetroNectin (TaKaRa)-coated plates. On day 5, CAR transduction efficiency was estimated as the percentage of GFP+ or mCherry+ live cells by flow cytometry. Mouse CAR-T cells were cultured in the presence of recombinant mouse IL-2 (PeproTech) at 30 IU/mL. Human CAR-T cells were cultured in the presence of recombinant human IL-7 (PeproTech) at 5 μg/mL and recombinant human IL-15 (PeproTech) at 5 μg/mL. For downstream experiments, CAR-T cell doses were normalized based on CAR gene transfer but not sorted to exclude CAR-negative T cells, resulting in varying total T cell dose.

**Flow Cytometry**

Cultured cells were washed twice with PBS and stained with a fixable viability dye (eFluor 450, #65-0863, Thermo Fisher Scientific). Surface staining was performed at 4 ºC using an Fc block (anti-mouse CD16/32, #14-0161-86, Thermo Fisher Scientific) and a mixture of antibodies: anti-mouse CD3e (APC, #100312, BioLegend), anti-mouse CD62L (PE/Cyanine7, #104418, BioLegend), and anti-mouse CD44 (APC-eFluor 780, #47-0441-82, Thermo Fisher Scientific) in MACS buffer containing 0.5% BSA (Miltenyi Biotec). Samples were analyzed using a BD FACSCantoII (BD Biosciences), and data were analyzed with FlowJo software (Tree Star).

**Multiplex Suspension Array**

Supernatants of culture medium, collected prior to flow cytometric analysis of mouse CAR-T cells, were analyzed using Bio-Plex Suspension Array System (BIO-RAD).

**Cytotoxicity assay**

Cytotoxicity assays were run on an xCELLigence RTCA (real-time cell analysis) DP instrument (ACEA Bioscience) according to the manufacturer’s instructions. Briefly, 3T3-mCD19-MYC or 3T3-hCD19-MYC cells were seeded at 10,000 cells per well in E-Plate. On the next day, mouse or human CAR-T cells were resuspended in fresh complete medium without cytokine support and added onto targeted cells at different Effector:Target ratios in the absence or presence of 100 nM TAK-981 and cell growth was monitored automatically.

**Mouse Model**

Female 6- to 8-week-old B6 mice were used for this study. Animal care and treatment followed institutional guidelines. For the syngeneic immunocompetent model, 1×10^6^ Eμ-Myc cells in 100 µL PBS were subcutaneously injected into the flank of B6 mice. In the TAK-981 monotherapy experiment (Supplementary Fig. S2), mice were treated with vehicle or 25 mg/kg TAK-981 via intraperitoneal injection twice weekly. In experiments using conventional preconditioning chemotherapy (PC), randomized mice were treated with 200 mg/kg cyclophosphamide and 20 mg/kg fludarabine via intraperitoneal injection. For TAK-981 combined with PC, 25 mg/kg TAK-981 was administered. For CAR-T cell infusion, 2×10^5^ CAR-T cells in 100 µL PBS were injected into the tail vein. For TAK-981 aid post-CAR-T treatment, 25 mg/kg TAK-981 was administered intraperitoneally once weekly for up to four doses. TAK-981 was dissolved in 20% HPbCD, 2.5% 1N HCl, 2.25% 1N NaOH, and 75.25% deionized water. Mice were monitored daily for body weight and general condition. Tumor volume was measured twice weekly using calipers and calculated as follows: length×width^2^×0.5. Mice were sacrificed when tumor volume reached 1,000 mm^3^ or due to health deterioration, in accordance with institutional guidelines.

**Statistical Analysis**

Group size was determined based on preliminary experimental results, and no statistical method was used to predetermine sample size. The indicated sample sizes (n) represent biological replicates. Statistical significance was determined using multiple t-tests with a false discovery rate cutoff value of 0.01, unpaired t-test, ordinary one-way ANOVA, or Kaplan-Meier method with log-rank test using GraphPad Prism 9. Significance levels are denoted as follows: *p < 0.05, **p < 0.01, ***p < 0.001, ****p < 0.0001, ns, not significant.

**Reference**

1. Li, G. *et al.* 4-1BB enhancement of CAR T function requires NF-κB and TRAFs. *JCI Insight* **3**, e121322 (2018).
2. Kotani, H. *et al.* Dual inhibition of SUMOylation and MEK conquers MYC-expressing KRAS-mutant cancers by accumulating DNA damage. *J Biomed Sci* **31**, 68 (2024).
